# Expectations and the Patient–Doctor Relationship: Ethical Considerations in a Case of Triploidy

**DOI:** 10.3390/healthcare13080912

**Published:** 2025-04-16

**Authors:** Iliya Mangarov, Irena Bradinova, Ralitsa Georgieva, Blagomir Zdravkov, Valentina Petkova, Irina Nikolova

**Affiliations:** 1 Department of Neonatology, Faculty of Medicine, Pediatric Hospital “Iv. Mitev”, Medical University of Sofia, 1612 Sofia, Bulgaria; 2 National Genetic Laboratory, Medical University of Sofia, 1431 Sofia, Bulgaria; 3 Department of Intensive Pediatric Unit, Pediatric Hospital “Iv. Mitev”, Medical University of Sofia, 1612 Sofia, Bulgaria; 4 Department of Organisation and Economics of Pharmacy, Faculty of Pharmacy, Medical University of Sofia, 1000 Sofia, Bulgaria; 5 Department of Pharmacology, Pharmacotherapy and Toxicology, Faculty of Pharmacy, Medical University of Sofia, 1000 Sofia, Bulgaria

**Keywords:** triploidy, live-born, DNA analysis, cytogenetic analysis, patient–doctor relationship, expecting parents

## Abstract

**Objectives**: Monitoring pregnancies is essential for community well-being. However, not all pregnancies progress normally, and some require termination. The objective was to emphasize the importance of trust in the doctor–patient relationship during this challenging time for expectant parents. **Case report**: During fetal morphology examination, parents were warned of a poor fetal prognosis, prompting a request for pregnancy termination. They consulted another specialist, who reassured them that the fetus appeared normal, though slightly hypotrophic. The child was born at 35 weeks gestational age and admitted to the neonatal ICU level III in an impaired general condition and polymalformative syndrome (triangular facies, epicanthic eyes, hypertelorism, retrognathia, low base of the nose, triangular mouth, lips angled downward, and small, dysplastic, and low-set earlobes). The child had syndactyly of fingers and toes. Cytogenetic analysis revealed a karyotype of 69, XX, +mar. The indirect DNA analysis revealed that the third gonosome is a Y chromosome. Death occurred 30 days post delivery, following severe dyspnea and bronchial obstruction, with desaturation and bradycardia. **Conclusions**: Triploid pregnancies are usually lost in the first trimester; however, very rarely, live births can occur. Hope for a positive outcome encouraged parents to continue the pregnancy, leading to a profoundly sorrowful experience and added strain on the healthcare system. Complex decisions put pressure on the patient–doctor relationship, as misplaced hope can impact both parties. Expectant parents facing difficult diagnoses require attentive support during this challenging time, grounded on a foundation of trust between doctor and patient.

## 1. Introduction

Chromosomal abnormalities are common, occurring in about 10% of all pregnancies. Triploidy is present in about 1% of pregnancies. Most pregnancies with triploid fetuses result in spontaneous termination during the first trimester [1,2,3]. Triploid pregnancies seldom result in a live-born child, with most dying within the first hours of life [4]. A few survive for days and even up to a month. The literature describes 13 cases of live-born children with triploidy who lived for a month [5,6,7,8,9,10,11,12,13,14,15,16,17].

Triploidy is a chromosomal disorder (69, XXX; 69, XXY; and 69, XYY) that occurs at conception. It can be triggered by both paternal (type I, referred to as diandric) and maternal (type II, referred to as digynic) origins. Diandrous triploidy most often arises when a single oocyte is fertilized by two spermatozoa [18,19], while digynous triploidy results from a complete failure of chromosome segregation during either the first or second meiotic division, leading to a diploid egg with two maternal sets of chromosomes [19,20]. Approximately two-thirds of diandrous triploids originate from dispermy, while the remaining one-third is attributable to meiotic errors that produce diploid sperm [21]. Many instances of diandrous triploidy are linked to partial hydatidiform moles; however, the mere presence of two paternal genomes is insufficient to induce molar development.

Infertile males carry a potential risk of transmitting chromosomally abnormal spermatozoa to oocytes through the process known as intracytoplasmic sperm injection (ICSI). The occurrence of diploidy, a commonly observed anomaly in sperm cells from males experiencing infertility, has been estimated to range from 0.2% to 9.6% [22,23,24]. Analysis of male pronuclei in tri-pronuclear zygotes produced by ICSI using sperm from oligospermic, cryptospermic, and azoospermic males revealed that 33.3% were diploid. In contrast, none of the pronuclei produced by normozoospermic males through in vitro fertilization (IVF) were diploid [25]. The data indicates that the majority of diandrous triploids generated by normozoospermic males occur as a result of dispermy, whereas those produced by oligozoospermic males arise from the utilization of diploid sperm. Increased maternal age has not been a factor in triploidy [26]. The digynic type accounts for most triploidy cases [27].

The 69, XXX and 69, XXY triploids occur with equal frequency, whereas the 69, XYY form is rarely observed, suggesting a significantly reduced viability of the 69, XYY triploid compared with the other two [19]. Common structural defects in both types of triploidy are facial abnormalities (low-set ears, hypertelorism, colobomata), syndactyly, simian palmar creases, microphallus, hypospadias, scrotal aplasia, cardiac anomalies, and hypoplasia of kidneys and adrenals [28,29,30].

During pregnancy monitoring, fetal morphology assessments are conducted at 11–13 weeks (early) or 19–23 weeks (late) of gestation. In inconclusive results, prenatal chromosome analysis or DNA testing should be performed [4,5]. It is important to note that these tests are expensive, not covered by the Bulgarian health insurance system, and are carried out only if the expectant parents choose to do so based on their doctor’s recommendation.

This article aims to present a case where neglecting the opinion of healthcare providers resulted in the birth of a child with 69, XXY triploidy. During fetal morphology examination (12 gestational weeks, g.w.), the parents were informed about the poor prognosis for the fetus due to intrauterine growth restriction and suspected aortic coarctation, prompting them to consider pregnancy termination. However, the couple consulted another fetal morphology specialist at 19 g.w., who reassured them that the fetus appeared morphologically normal, although slightly hypotrophic. The presentation of two relatively opposing diagnoses, along with the desire for a healthy baby, resulted in ignoring the unfavorable diagnosis and the consequent unfortunate outcome of giving birth to a child that is incompatible with life.

## 2. Case Report

A child was born on 17 December 2020, at 35 g.w. via cesarean section. The pregnancy resulted from normal conception by a seemingly healthy Caucasian couple: a 33-year-old mother and a 42-year-old father. This was the family’s second pregnancy, following a normal first one. The pregnancy was monitored at several private hospitals, which is a prerequisite for obtaining incomplete information on the course and follow-up of the pregnancy.

The child was born in a moderately depressed state, with an Apgar score of 6 at both the 1st and 5th min. The amniotic fluid was clear. The infant weighed 1300 g, measured 39 cm in length, and had a head circumference of 28 cm (under the 3rd percentile). Notably, the child displayed features consistent with a polymalformative syndrome, characterized by a triangular facial structure, epicanthic folds, hypertelorism, retrognathia, a low nasal base, and a downward-angled triangular mouth. Additionally, the earlobes were small, dysplastic, and positioned low on the head. Syndactyly was present between the second and third toes of both feet and between the third and fourth fingers of both hands. Partial syndactyly was also noted between the second and third fingers, limited to the bases (proximal phalanges) of the left hand. A four-fingered groove was observed bilaterally. Primary resuscitation was conducted in the delivery room. The infant was placed on duo positive airway pressure (DUOPAP); however, due to a compromised condition and persistent tachycardia after delivery, the child was transferred to a neonatal ICU level III for further diagnostic evaluation and treatment on 18 December 2020. The newborn was classified as female due to the absence of apparent male genitalia. Generally, aneuploidies, such as trisomy 13 and triploidy, are associated with cases of ambiguous genitalia [31].

Upon admission, the patient exhibited an impaired general condition but was afebrile. The skin appeared marmorated, and the mucous membranes were pale pink. Notably, aplasia cutis congenita was noted in the vertex region of the head. The examination revealed a regular head shape, with the anterior fontanelle measuring 30 mm by 30 mm, while the posterior fontanelle remained open and appeared fused with the anterior fontanelle. The cranial sutures were not fused. The chest configuration was regular and symmetrical. Auscultation detected bilateral attenuated vesicular breath sounds, accompanied by exudative findings and mild to moderate dyspnea. The respiratory rate was 50 breaths per minute, and transcutaneous oxygen saturation was at 90%, with a fraction of inspired oxygen (FiO_2_) of 21%. The cardiac examination indicated a rhythmic heartbeat with clear tones, and the heart rate ranged from 130 to 150 beats per minute. A consultation with a pediatric cardiologist revealed that there is no underlying cardiopathy present. Central pressure was measured at 40 mmHg, with peripheral pulsations present. No abnormalities of the gastrointestinal tract, diaphragmatic hernia, or hepatosplenomegaly were observed. The neurological assessment revealed an abnormal state characterized by lethargy progressing to soporific behavior, along with generalized muscular hypotonia. There was a decrease in spontaneous and evoked activity, but no abnormal movements were noted. Additionally, Moreau’s reflex was positive in the first phase. The examination of the cerebrocranial and peripheral nerves seemed normal. In terms of the urogenital system, there was evidence of poor differentiation, suggesting clitoromegaly or micropenis. The vaginal introitus was extremely narrow, and small labia were absent. Diuresis and defecation occurred spontaneously. Both feet displayed a rocker-bottom deformity, and marked hypermobility in the hip joints was evident in this case.

An echocardiogram performed upon admission showed unremarkable results. Due to the worsening respiratory failure, the patient was intubated and placed on a ventilator using Pressure Control–Assist Control (PC-AC) mode with intensive ventilatory parameters. Isogroup hemotransfusion of erythrocyte concentrate was administered over two consecutive days (11 January and 12 January) due to low hemoglobin levels (102 g/L) and evidence of high oxygen needs.

A microbiological examination of a hemoculture conducted on 23 December 2020, revealed no growth. However, an analysis of a tracheal aspirate on 4 January 2021, revealed the presence of Staphylococcus epidermidis. The isolate displayed resistance to amoxicillin–clavulanic acid, cefazolin, cefotaxime, ceftriaxone, cefuroxime (sodium), chloramphenicol, ciprofloxacin, gentamicin, levofloxacin, meropenem, penicillin, trimethoprim–sulfamethoxazole, and ampicillin. In contrast, it demonstrated susceptibility to azithromycin, clarithromycin, clindamycin, erythromycin, linezolid, and vancomycin.

The X-ray findings indicated that the left lung was entirely homogeneously shaded, with only the pulmonary apex and the phrenicocostal sinus remaining visible. This observation may suggest the presence of pneumonia and/or atelectasis. Furthermore, homogeneous shadowing was noted in the lower right paracardial lobe. The lateral outline of this shadowing is straight and well defined, implying possible subatelectasis. Importantly, the hilar shadows exhibited no signs of enlargement.

Therapeutic interventions included mechanical ventilation in PC-AC mode and infusions of glucose–saline, amino acids, antibiotics, diuretics, and corticosteroids. Vaccinations have not been performed. The antibiotic regimen included Cefepime (50 mg/kg/dose q8h) prescribed for 21 days until 31 December. Ampicillin (75 mg/kg/dose q12h) and amikacin (7.5 mg/kg/dose q12h) were administered for 4 days until 22 December. Due to elevated inflammatory activity (CRP 55.96 mg/L), antibiotic therapy was revised, and vancomycin (15 mg/kg/dose q8h) and meropenem (20 mg/kg/dose q8h) were initiated on December 22 and continued until 8 January. Sulperazon (sulbactam/cefoperazone) (50 mg/kg/dose q12h) was administered from 9 January to 15 January.

Two weeks after admission, the patient remained in a severely compromised general condition, requiring mechanical ventilation and supplemental oxygen due to significant dyspnea, reduced respiratory function, exudative manifestations, and bronchial obstruction. The patient continued to depend on the ventilator for breathing support. Neurologically, the child exhibited signs of lethargy and impaired consciousness; however, there were intermittent responses to painful stimuli and a mild improvement in spontaneous motor activity. Given the elevated inflammatory markers, the antibiotic regimen was re-evaluated, resulting in positive outcomes. The patient continued to produce copious secretions from the endotracheal tube. A respiratory assessment revealed diminished vesicular sounds along with exudative findings. The patient tolerated enteral feedings of up to 8–10 mL of preNAN. Additionally, adequate spontaneous diuresis (70–90 mL) and regular bowel movements were observed.

On 15 January 2021, the patient’s condition significantly worsened, exhibiting acute shortness of breath, severe bronchial obstruction, low oxygen saturation, and a slow heart rate. Despite the use of full-volume resuscitation measures, these interventions proved ineffective. Unfortunately, the patient passed away that same day. The parents decided against having an autopsy performed.

### 2.1. Laboratory Findings

Upon admission on 18 December 2020, routine blood tests indicated low hemoglobin levels (118 g/L) and low glucose levels (2.1 mmol/L), with an RDW-CV (a red cell distribution width test) of 1.6%. Mean Corpuscular Hemoglobin (MCH) and Mean Corpuscular Volume (MCV) levels were also elevated, recorded at 49.1 pg and 143 fL, respectively. C-reactive protein was significantly elevated at 55.96 mg/L (normal < 5 mg/L). The clinical course was complicated by metabolic acidosis, which was corrected through sodium bicarbonate infusion. Hormone tests conducted on 7 January 2021, revealed a high prolactin level of 1397 mIU/L (normal range: 40–530 mIU/L), while levels of thyroid hormones, testosterone, growth hormone, luteinizing hormone, follicle-stimulating hormone, and cortisol remained within normal limits. Antibody tests performed on 21 December 2020, for rubella, cytomegalovirus, herpes simplex, and toxoplasmosis returned negative results.

### 2.2. Cytogenetic Analysis

A cytogenetic analysis of peripheral blood lymphocytes and skin fibroblasts revealed a pathological karyotype of 69, XX, +mar, indicating a triploid chromosomal aberration. The third gonosome lacked the structure of an X chromosome. In size and appearance, the identified chromosome resembled a Y chromosome or a deleted X chromosome (del Xq). A blood sample was also sent for further indirect DNA analysis of chromosomal disorders using Quantitative Fluorescent Polymerase Chain Reaction (QF PCR). The QF PCR analysis confirmed the triploid profile (three copies of all included chromosomes) in the patient’s blood sample, revealing the presence of a Y chromosome corresponding to the XXY karyotype.

## 3. Discussion

Most triploid live-born cases survive only for hours to a few days after birth. Recently, Walsh and Sharma [5] published a review of 13 case reports of live-born individuals with triploidy who survived 30 days or more. Our patient survived for 30 days. Triploidy was confirmed through cytogenetic analysis and further DNA testing. Live-born triploid newborns have well-recognized anomalies, such as eye defects, dysplastic cranial bones, low-set ears, joint hypermobility, syndactyly, skeletal abnormalities, genital malformations, and hypertelorism, as evident in this case as well.

Our case also raises some moral issues. Based on data from an ultrasound assessment of fetal morphology performed at 12 g.w., the parents were warned about the necessity of terminating the pregnancy. Nevertheless, the couple chose to consult an independent certified fetal morphology specialist at 19 g.w., who assured them of a normally developing pregnancy but noted that the fetus was hypotrophic. This solitary medical opinion and the hope for a positive outcome, while overlooking the warnings from the initial fetal morphology examination, influenced the parents’ decision to proceed with the pregnancy. Their distrust of doctors advocating for immediate termination and their dismissal of warnings resulted in devastating emotional and physical experiences for the mother, placing a burden on the healthcare system [32,33].

The patient–physician relationship is a fundamental healthcare concept involving vulnerability and trust. It represents one of the most significant and meaningful experiences humans can share, influenced by various subjective and objective factors [34,35,36]. This process does not always yield positive outcomes for both parties [37]. The necessity of making difficult decisions by the patient or physician can affect this delicate relationship. On one hand, the desire for a favorable outcome may mislead patients into making the right decision, which discourages doctors who, lacking trust in them, may lose the willingness and motivation to support the patient’s decision [38,39]. Therefore, this relationship and the interactions that result from it are not always ideal. Special consideration should be given to expecting parents and their decision-making in cases of unfavorable fetal diagnosis and the need for pregnancy termination [32]. The guiding principle is that these individuals are relatively young and healthy, yet their emotional state is highly fragile and vulnerable. As pregnancy is filled with hope and expectations for a healthy baby, many support groups share stories of misdiagnosis that have led to the birth of healthy children, contrary to the assurances given by healthcare professionals. Family members, friends, the internet, and patient advocacy groups may unintentionally cause confusion by offering empathy and support to expectant parents [40]. When faced with the difficult decision to terminate a pregnancy, it is crucial to have the diagnosis confirmed. In the case presented here, the diagnosis was not verified through the second fetal morphology, resulting in even greater grief for the hopeful parents-to-be. A prenatal chromosome analysis/DNA test was not performed because it is very expensive and not covered by health insurance. During the NICU treatment period, a strong and trustworthy relationship was established with the parents. The unfavorable prognosis was elucidated, and they recognized their erroneous decision. The parents received unwavering support during the month their child was alive, even though it was a devastating time for them.

## 4. Conclusions

Losing a child is a tragedy. However, when it is linked to a genetic abnormality, the likelihood of a woman hesitating to give birth again is rather high. The presented case raises numerous moral issues, such as the failure of health professionals to establish sufficient trust with the expectant parents or to offer adequate support for their difficult decision. In our case, the presence of two relatively opposite diagnoses, the neglect of the unfavorable diagnosis, and the hope for a favorable outcome led to the wrong decision to continue the pregnancy. In conclusion, several factors have influenced this case: on one side are the parents, on the other side are the health care providers, and on the third side is the health insurance system.

## Data Availability

Data are contained within the article.
